# Association between gut microbiota and gastrointestinal cancer: a two-sample bi-directional Mendelian randomization study

**DOI:** 10.3389/fmicb.2023.1181328

**Published:** 2023-07-18

**Authors:** Qing Su, Chen Jin, Zhiyuan Bo, Yi Yang, Jingxian Wang, Juejin Wang, Junxi Zhou, Yaqing Chen, Hao Zeng, Gang Chen, Yi Wang

**Affiliations:** ^1^Department of Epidemiology and Biostatistics, School of Public Health and Management, Wenzhou Medical University, Wenzhou, China; ^2^Department of Epidemiology, Center for Global Health, School of Public Health, Nanjing Medical University, Nanjing, China; ^3^Department of Hepatobiliary Surgery, The First Affiliated Hospital of Wenzhou Medical University, Wenzhou, Zhejiang, China

**Keywords:** gut microbiota, gastrointestinal cancer, Mendelian randomization, instrumental variable, causal relationship

## Abstract

**Background:**

The gut microbiome is closely related to gastrointestinal (GI) cancer, but the causality of gut microbiome with GI cancer has yet to be fully established. We conducted this two-sample Mendelian randomization (MR) study to reveal the potential causal effect of gut microbiota on GI cancer.

**Materials and methods:**

Summary-level genetic data of gut microbiome were derived from the MiBioGen consortium and the Dutch Microbiome Project. Summary statistics of six GI cancers were drawn from United Kingdom Biobank. Inverse-variance-weighted (IVW), MR-robust adjusted profile score (MR-RAPS), and weighted-median (WM) methods were used to evaluate the potential causal link between gut microbiota and GI cancer. In addition, we performed sensitivity analyses and reverse MR analyses.

**Results:**

We identified potential causal associations between 21 bacterial taxa and GI cancers (values of *p* < 0.05 in all three MR methods). Among them, phylum *Verrucomicrobia* (OR: 0.17, 95% CI: 0.05–0.59, *p* = 0.005) retained a strong negative association with intrahepatic cholangiocarcinoma after the Bonferroni correction, whereas order *Bacillales* (OR: 1.67, 95% CI: 1.23–2.26, *p* = 0.001) retained a strong positive association with pancreatic cancer. Reverse MR analyses indicated that GI cancer was associated with 17 microbial taxa in all three MR methods, among them, a strong inverse association between colorectal cancer and family *Clostridiaceae1* (OR: 0.91, 95% CI: 0.86–0.96, *p* = 0.001) was identified by Bonferroni correction.

**Conclusion:**

Our study implicates the potential causal effects of specific microbial taxa on GI cancer, potentially providing new insights into the prevention and treatment of GI cancer through specific gut bacteria.

## 1. Introduction

In 2020, the five major types of gastrointestinal (GI) cancer (including esophageal, gastric, colorectal, liver, and pancreatic cancers) account for 25.8% of the global cancer incidence and 35.4% of the global cancer-related deaths (Sung et al., 2021). GI cancers are significant contributors to the global burden of cancer and pose a serious challenge to public health (Arnold et al., 2020), so finding the etiology and applying suitable preventive measures are urgent.

There is increasing evidence that intestinal microbiota is closely related to GI cancer (Tong et al., 2021). Intestinal flora is involved in the occurrence and progression of colorectal cancer (CRC) by affecting the inflammatory process in the intestine and producing metabolites (Brennan and Garrett, 2016; Wong and Yu, 2019). Epidemiological studies have shown that the microbial composition differs between CRC patients and healthy controls, and may serve as biomarkers for CRC screening and prognosis (Wong and Yu, 2019). The intestinal microbiota not only influences the occurrence of CRC via local effects but also has long-distance effects on other cancers, for example, affecting the development of liver cancer through the gut–liver axis (Schwabe and Jobin, 2013; Yu and Schwabe, 2017). Intestinal microbial metabolites and microbial components can be transferred to the liver through the gut–liver axis (Ohtani and Hara, 2021). In addition, abundance differences in gut microbes between patients with other GI cancers (e.g., pancreatic, esophageal, and gastric cancers) and healthy controls were also found (Yu et al., 2021; Cheung et al., 2022; Kartal et al., 2022). However, the causality of gut microbiota with GI cancer has not been fully established due to the potential effects of residual confounding and reverse causality.

Mendelian randomization (MR), a common method for examining causal relationships between exposures and outcomes, has been used to explore potential causal associations between gut microbiota and multiple diseases (Li et al., 2022, 2023). Recently, using two-sample MR analysis, two studies reported the potential causal association between intestinal microbiota and CRC and one study reported the potential causality of gut microbiota with gastric cancer (Ni et al., 2022; Long et al., 2023). In addition, there was an MR analysis exploring the relationship of 57 bacterial taxa (including four phyla, eight classes, six orders, 10 families, and 29 genera) with liver cancer (Ma et al., 2023). However, potential causal associations of many other gut microbial taxa with liver cancer are unknown, and the potential causal relationship of gut microbiome with pancreatic and esophageal cancer is not well established. Therefore, this study performed two-sample MR analysis using the genome-wide association study (GWAS) datasets containing 211 bacterial taxa at the phylum to genus level from the MiBioGen consortium (Kurilshikov et al., 2021) and 105 bacterial taxa at the species level from the Dutch Microbiome Project (Lopera-Maya et al., 2022) to reveal the potential causal relationships of gut microbiota with esophageal, gastric, colorectal, liver, and pancreatic cancers.

## 2. Materials and methods

### 2.1. Study design

**Figure 1:**
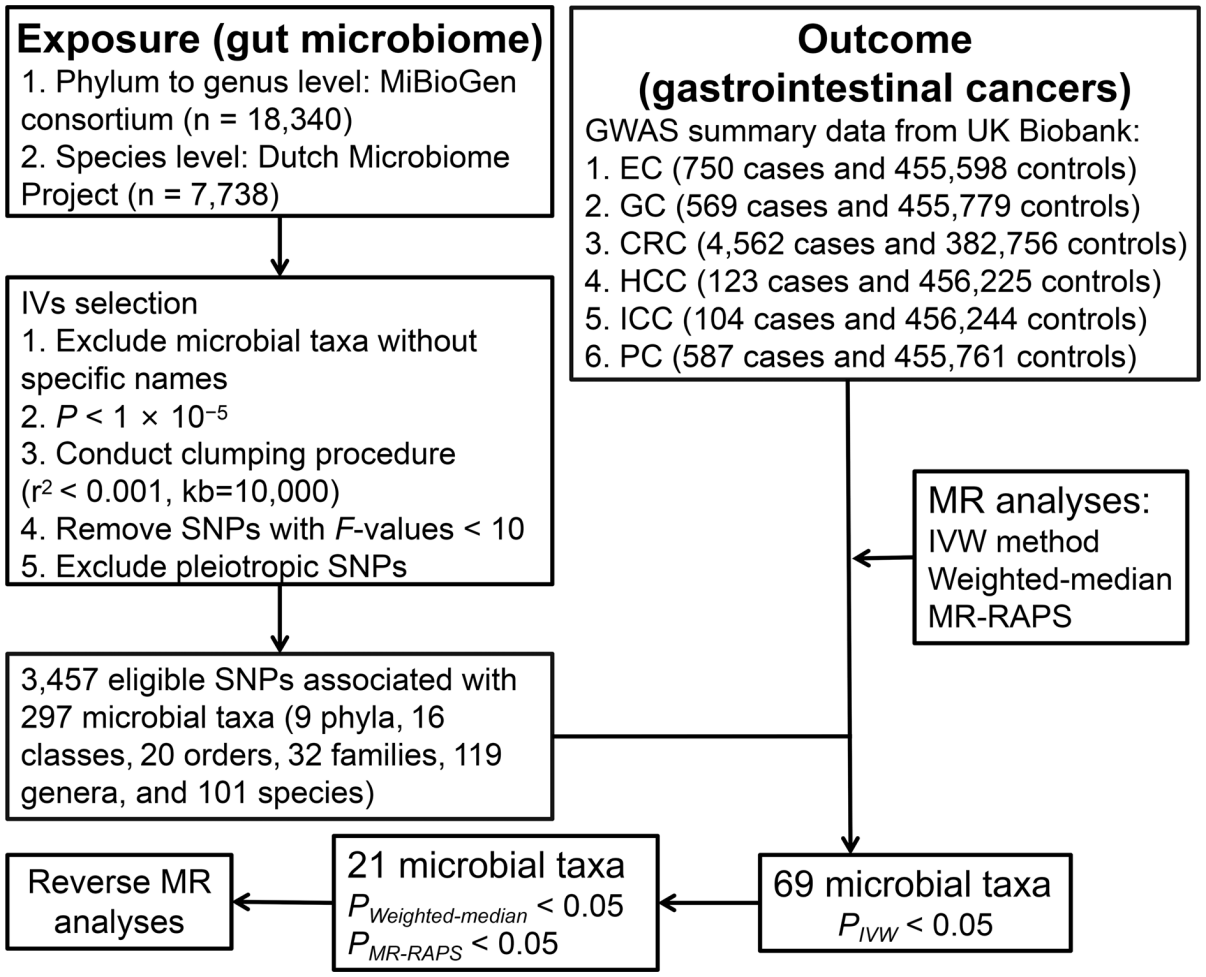
The study design and workflow of the present MR study. EC, esophageal cancer; GC, gastric cancer; CRC, colorectal cancer; HCC, hepatocellular carcinoma; ICC, intrahepatic cholangiocarcinoma; PC, pancreatic cancer; IVs, instrumental variables; MR, Mendelian randomization; SNP, single-nucleotide polymorphism; IVW, Inverse-variance-weighted; and MR-RAPS, MR-robust adjusted profile score.

The study design of the present two-sample MR analysis is shown in Figure 1. To reliably infer the potential causality of gut microbiome with GI cancer risk using MR approach, we tried to meet three key assumptions of MR analysis. First, the instrumental variables (IVs) are correlated with gut microbiome. Second, IVs are unrelated to confounders influencing this association. Third, IVs influence the GI cancer risk only through gut microbiota (Davies et al., 2018).

### 2.2. Data sources and instruments selection

The genetic data of human gut microbiome at the phylum to genus level were obtained from the multi-ethnic MiBioGen consortium comprising 24 population-based cohorts with 18,340 participants. A total of 211 gut microbial taxa were included in this GWAS dataset, of which 15 were unknown families or genera and were excluded, leaving 196 microbial taxa for MR analysis. The summary statistics on the species level of gut microbiota were derived from the Dutch Microbiome Project including a total of 105 species with 7,738 participants of European ancestry. To obtain more comprehensive results, IVs that attained locus-wide significance (*p* < 1 × 10^−5^) were selected. In parallel, single-nucleotide polymorphisms (SNPs) in linkage disequilibrium were excluded by the PLINK clumping method (*r*^2^ < 0.001, kb = 10,000). Then, SNPs with *F*-statistics [formula: *R*^2^/K × (N − K − 1)/(1 − *R*^2^)] < 10 were removed (Palmer et al., 2012). Finally, we searched the PhenoScanner website for additional phenotypes associated with gut microbiota-related SNPs and removed SNPs associated with confounders (body mass index, waist circumference, smoking, alcohol intake, blood pressure, blood lipids, coronary artery disease, weight, hip circumference, fat percentage, diabetes, worrier or anxious feelings, nervous feelings, chronotype, birth weight, hypothyroidism, gout, Gamma glutamyl transferase, skin cancer, ovarian cancer, malabsorption or coeliac disease, primary biliary cholangitis, and Hodgkin’s disease). Four species with less than three available SNPs were excluded. A total of 297 bacterial taxa were included in the MR analysis.

Genome-wide association study summary statistics for esophageal cancer (750 cases and 455,598 controls), gastric cancer (569 cases and 455,779 controls), CRC (4,562 cases and 382,756 controls), hepatocellular carcinoma (HCC, 123 cases and 456,225 controls), intrahepatic cholangiocarcinoma (ICC, 104 cases and 456,244 controls), and pancreatic cancer (587 cases and 455,761 controls) were obtained from United Kingdom Biobank, with details described elsewhere (Zhou et al., 2018; Jiang et al., 2021). No additional ethics approval or informed consent was required due to our study was based on public databases.

### 2.3. Statistical analysis

The potential causality of gut microbiota and GI cancer risk was primarily calculated by inverse-variance-weighted (IVW) method. Cochran’s Q test was used for assessment of heterogeneity, using random-effects IVW when heterogeneity was significant (*p* < 0.05) and, conversely, fixed-effects IVW. The consistency of results was examined by two additional approaches: MR-robust adjusted profile score (MR-RAPS) method and weighted-median (WM) method. The condition for WM method to obtain consistent estimates of causal effects is that half of SNPs are valid IVs (Bowden et al., 2016). MR-RAPS can make robust inferences when it contains weak IVs (Zhao et al., 2019). To assess pleiotropy, we performed MR-Egger and MR-Pleiotropy RESidual Sum and Outlier (MR-PRESSO) tests (Bowden et al., 2015; Verbanck et al., 2018), the latter of which could also detect outliers and test for differences in results before and after eliminating outliers (Verbanck et al., 2018). In addition, leave-one-out analysis was conducted to assess the impact on overall estimates by a single SNP.

To obtain a more rigorous explanation of causality, we used Bonferroni method to establish multiple testing significance thresholds at different taxonomic levels separately based on the number of bacteria under each taxonomic level [5.6 × 10^−3^ (0.05/9) for phylum, 3.1 × 10^−3^ (0.05/16) for class, 2.5 × 10^−3^ (0.05/20) for order, 1.6 × 10^−3^ (0.05/32) for family, 4.2 × 10^−4^ (0.05/119) for genus, and 5.0 × 10^−4^ (0.05/101) for species]. *P* values reaching nominal significance (*p* < 0.05) were considered to have nominal potential causal effects. Finally, reverse MR analyses utilizing SNPs correlated with GI cancer (*p* < 5 × 10^−6^) as IVs were performed to examine whether GI cancer had a causal impact on gut microbial taxa. “TwoSampleMR” and “MR-PRESSO” packages were used for analyses in R program (version 4.2.2).

## 3. Results

### 3.1. Overview

**Figure 2:**
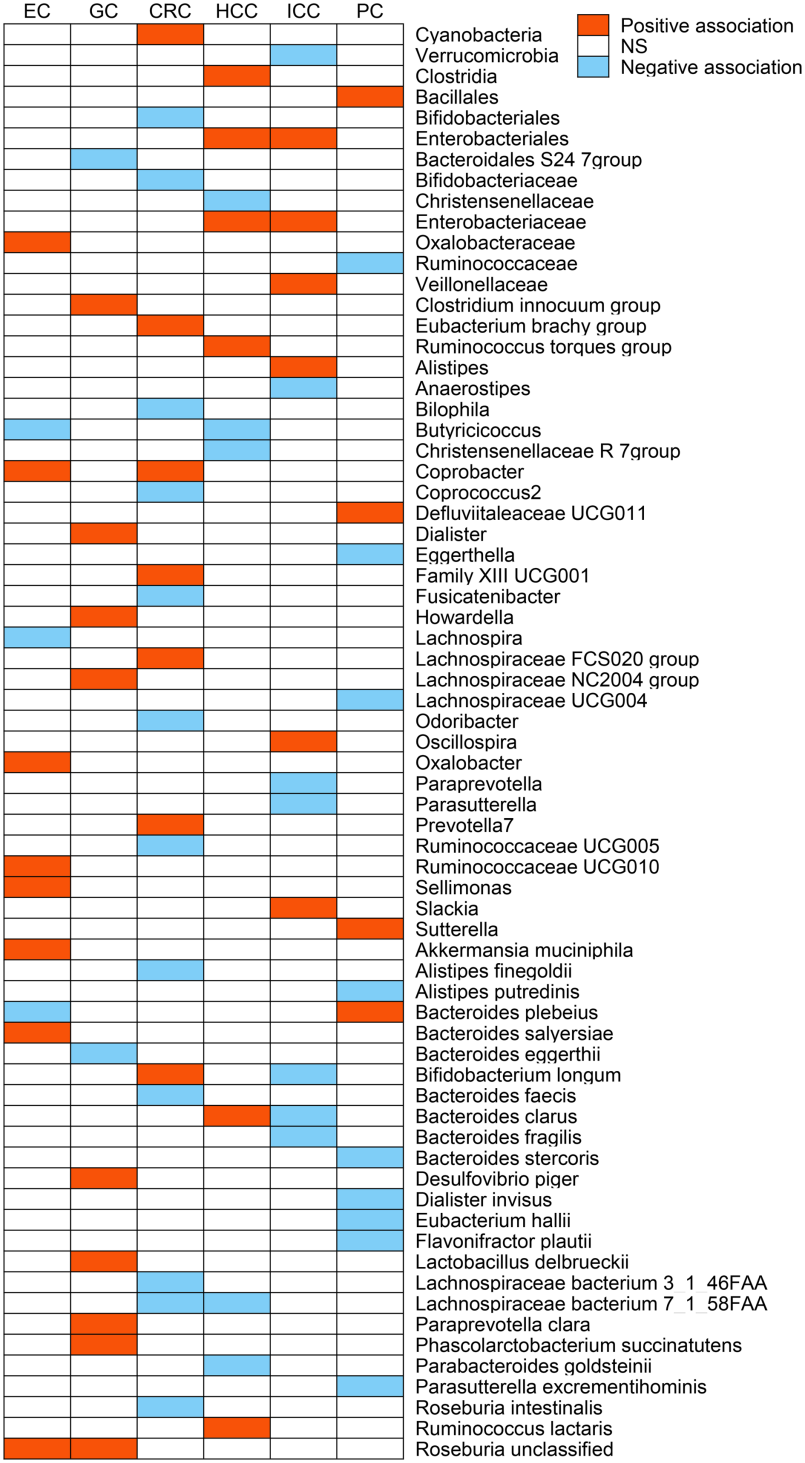
The potential causal relationship between gut microbiome and gastrointestinal (GI) cancer risk in the IVW method (*p* < 0.05). Red represents the risk taxa for GI cancer, blue represents the protective taxa for GI cancer, and white represents no causal taxa for GI cancer. EC, esophageal cancer; GC, gastric cancer; CRC, colorectal cancer; HCC, hepatocellular carcinoma; ICC, intrahepatic cholangiocarcinoma; PC, pancreatic cancer; and NS, no significant association.

After a series of IV screening steps, a total of 3,457 eligible SNPs from 297 microbial taxa were finally included in this analysis. Details of IVs are listed in Supplementary Table S1. The IVW method identified 69 microbial taxa associated with esophageal cancer, gastric cancer, CRC, HCC, ICC, or pancreatic cancer (Figure 2). However, only 21 microbial taxa remained stable in both WM and MR-RAPS methods (Table 1). The scatter plots of the associations of these 21 microbial taxa with the corresponding GI cancers are shown in Supplementary Figures S1–S6. And the statistical power of these microbial taxa calculated by an online tool^1^ is presented in Table 1.

**Table 1:** Causal associations between genetically predicted 21 microbial taxa and gastrointestinal cancer risk.

Outcome	Microbial taxa (Exposure)	No. of SNP	MR method	OR (95% CI)	*p* value	Statistical power
EC	*Oxalobacteraceae*	15	IVW	1.33 (1.01–1.77)	0.045	65%
		15	WM	1.52 (1.03–2.23)	0.033	
		15	MR-RAPS	1.45 (1.07–1.97)	0.016	
EC	*Oxalobacter*	12	IVW	1.43 (1.07–1.92)	0.016	84%
		12	WM	1.48 (1.01–2.18)	0.046	
		12	MR-RAPS	1.47 (1.07–2.01)	0.017	
EC	*Ruminococcaceae UCG010*	8	IVW	2.54 (1.39–4.64)	0.002	100%
		8	WM	2.32 (1.02–5.28)	0.044	
		8	MR-RAPS	2.61 (1.35–5.03)	0.004	
GC	*Howardella*	11	IVW	1.69 (1.21–2.34)	0.002	98%
		11	WM	1.96 (1.25–3.08)	0.003	
		11	MR-RAPS	1.73 (1.21–2.49)	0.003	
GC	*Roseburia unclassified*	13	IVW	1.34 (1.03–1.74)	0.027	71%
		13	WM	1.43 (1.00–2.03)	0.050	
		13	MR-RAPS	1.38 (1.04–1.84)	0.025	
CRC	*Bilophila*	16	IVW	0.79 (0.66–0.95)	0.012	63%
		16	WM	0.75 (0.58–0.98)	0.033	
		16	MR-RAPS	0.78 (0.63–0.97)	0.025	
CRC	*Lachnospiraceae FCS020 group*	15	IVW	1.30 (1.08–1.57)	0.005	91%
		15	WM	1.30 (1.01–1.68)	0.039	
		15	MR-RAPS	1.32 (1.08–1.60)	0.007	
CRC	*Prevotella7*	12	IVW	1.19 (1.06–1.33)	0.003	93%
		12	WM	1.22 (1.04–1.43)	0.014	
		12	MR-RAPS	1.21 (1.07–1.38)	0.003	
HCC	*Butyricicoccus*	9	IVW	0.22 (0.06–0.79)	0.021	25%
		9	WM	0.16 (0.03–0.99)	0.048	
		9	MR-RAPS	0.20 (0.05–0.83)	0.027	
HCC	*Ruminococcus lactaris*	4	IVW	4.79 (1.46–15.74)	0.010	100%
		4	WM	5.08 (1.04–24.76)	0.044	
		4	MR-RAPS	5.37 (1.01–28.69)	0.049	
ICC	*Verrucomicrobia*	12	IVW	0.17 (0.05–0.59)	0.005	23%
		12	WM	0.17 (0.03–0.92)	0.040	
		12	MR-RAPS	0.16 (0.04–0.60)	0.007	
ICC	*Enterobacteriales*	10	IVW	5.63 (1.16–27.43)	0.032	100%
		10	WM	7.31 (1.01–53.03)	0.049	
		10	MR-RAPS	6.48 (1.18–35.45)	0.031	
ICC	*Enterobacteriaceae*	10	IVW	5.63 (1.16–27.43)	0.032	100%
		10	WM	7.31 (1.01–52.97)	0.049	
		10	MR-RAPS	6.48 (1.18–35.45)	0.031	
ICC	*Veillonellaceae*	21	IVW	3.58 (1.29–9.93)	0.014	100%
		21	WM	4.23 (1.04–17.20)	0.044	
		21	MR-RAPS	3.93 (1.33–11.65)	0.014	
ICC	*Paraprevotella*	13	IVW	0.27 (0.11–0.67)	0.005	31%
		13	WM	0.24 (0.07–0.86)	0.029	
		13	MR-RAPS	0.25 (0.09–0.68)	0.007	
ICC	*Bacteroides clarus*	7	IVW	0.52 (0.28–0.99)	0.046	28%
		7	WM	0.32 (0.13–0.79)	0.013	
		7	MR-RAPS	0.43 (0.21–0.87)	0.019	
PC	*Bacillales*	11	IVW	1.67 (1.23–2.26)	0.001	99%
		11	WM	1.58 (1.04–2.40)	0.031	
		11	MR-RAPS	1.77 (1.27–2.48)	<0.001	
PC	*Eggerthella*	11	IVW	0.63 (0.43–0.93)	0.020	47%
		11	WM	0.59 (0.35–0.99)	0.044	
		11	MR-RAPS	0.63 (0.41–0.95)	0.027	
PC	*Sutterella*	12	IVW	2.45 (1.38–4.37)	0.002	100%
		12	WM	2.54 (1.16–5.58)	0.020	
		12	MR-RAPS	2.54 (1.36–4.76)	0.003	
PC	*Flavonifractor plautii*	6	IVW	0.57 (0.40–0.82)	0.002	69%
		6	WM	0.53 (0.32–0.88)	0.015	
		6	MR-RAPS	0.55 (0.36–0.85)	0.006	
PC	*Eubacterium hallii*	12	IVW	0.61 (0.46–0.83)	0.001	74%
		12	WM	0.61 (0.40–0.92)	0.019	
		12	MR-RAPS	0.61 (0.44–0.84)	0.003	

Presented are gut microbial taxa that were statistically significant in all MR analyses (IVW, WM, and MR-RAPS). EC, esophageal cancer; GC, gastric cancer; CRC, colorectal cancer; HCC, hepatocellular carcinoma; ICC, intrahepatic cholangiocarcinoma; PC, pancreatic cancer; MR, Mendelian randomization; SNP, single-nucleotide polymorphism; IVW, inverse-variance weighted; WM, weighted median; MR-RAPS, MR-robust adjusted profile score; OR, odds ratio; and CI, confidence interval.

### 3.2. Esophageal cancer

The IVW analysis indicated that *Oxalobacteraceae* (OR: 1.33, 95% CI: 1.01–1.77), *Coprobacter* (OR: 1.60, 95% CI: 1.15–2.21), *Oxalobacter* (OR: 1.43, 95% CI: 1.07–1.92), *Ruminococcaceae UCG010* (OR: 2.54, 95% CI: 1.39–4.64), *Sellimonas* (OR: 1.46, 95% CI: 1.07–1.99), *Akkermansia muciniphila* (OR: 1.57, 95% CI: 1.13–2.17), *Bacteroides salyersiae* (OR: 1.24, 95% CI: 1.01–1.52), and *Roseburia unclassified* (OR: 1.33, 95% CI: 1.06–1.68) were correlated with increased esophageal cancer risk (*p* < 0.05), whereas *Butyricicoccus* (OR: 0.56, 95% CI: 0.33–0.94), *Lachnospira* (OR: 0.44, 95% CI: 0.21–0.93), and *Bacteroides plebeius* (OR: 0.72, 95% CI: 0.57–0.92) were related to a reduced risk of esophageal cancer (*p* < 0.05; Figure 2). However, only *Oxalobacteraceae*, *Oxalobacter*, and *Ruminococcaceae UCG010* maintained consistent results in both WM and MR-RAPS methods (Table 1). Leave-one-out analysis of these three bacterial taxa found that some SNPs of *Oxalobacteraceae* and *Oxalobacter* might dominate the positive results (Supplementary Figure S7).

### 3.3. Gastric cancer

According to IVW method (Figure 2), *Bacteroidales S24 7group* (OR: 0.56, 95% CI: 0.34–0.90) and *Bacteroides eggerthii* (OR: 0.69, 95% CI: 0.49–0.97) were associated with a lower risk of gastric cancer (*p* < 0.05), while *Clostridium innocuum group* (OR: 1.48, 95% CI: 1.04–2.10), *Dialister* (OR: 1.85, 95% CI: 1.06–3.25), *Howardella* (OR: 1.69, 95% CI: 1.21–2.34), *Lachnospiraceae NC2004 group* (OR: 1.72, 95% CI: 1.10–2.70), *Paraprevotella clara* (OR: 1.48, 95% CI: 1.10–2.00), *Lactobacillus delbrueckii* (OR: 1.18, 95% CI: 1.01–1.38), *Phascolarctobacterium succinatutens* (OR: 1.33, 95% CI: 1.01–1.75), *Desulfovibrio piger* (OR: 1.55, 95% CI: 1.02–2.35), and *Roseburia unclassified* (OR: 1.34, 95% CI: 1.03–1.74) were correlated with higher gastric cancer risk (*p* < 0.05). Of these, only *Howardella* and *Roseburia unclassified* remained stable in WM and MR-RAPS methods (Table 1), and SNPs with significant effects were not identified in *Howardella* but in *Roseburia unclassified* by leave-one-out analysis (Supplementary Figure S8).

### 3.4. Colorectal cancer

*Bifidobacteriales* (OR: 0.75, 95% CI: 0.62–0.91), *Bifidobacteriaceae* (OR: 0.75, 95% CI: 0.62–0.91), *Bilophila* (OR: 0.79, 95% CI: 0.66–0.95), *Coprococcus2* (OR: 0.80, 95% CI: 0.65–0.99), *Fusicatenibacter* (OR: 0.82, 95% CI: 0.68–0.99), *Odoribacter* (OR: 0.73, 95% CI: 0.57–0.94), *Ruminococcaceae UCG005* (OR: 0.81, 95% CI: 0.67–0.98), *Alistipes finegoldii* (OR: 0.84, 95% CI: 0.71–0.99), *Bacteroides faecis* (OR: 0.93, 95% CI: 0.88–1.00), *Lachnospiraceae bacterium 3_1_46FAA* (OR: 0.83, 95% CI: 0.70–0.98), *Lachnospiraceae bacterium 7_1_58FAA* (OR: 0.85, 95% CI: 0.73–1.00), and *Roseburia intestinalis* (OR: 0.84, 95% CI: 0.72–0.98) were negatively correlated with CRC risk in the IVW approach (*p* < 0.05; Figure 2). As for *Cyanobacteria* (OR: 1.19, 95% CI: 1.01–1.40), *Eubacterium brachy group* (OR: 1.17, 95% CI: 1.03–1.31), *Coprobacter* (OR: 1.16, 95% CI: 1.02–1.32), *Family XIII UCG001* (OR: 1.24, 95% CI: 1.00–1.54), *Lachnospiraceae FCS020 group* (OR: 1.30, 95% CI: 1.08–1.57), *Prevotella7* (OR: 1.19, 95% CI: 1.06–1.33), and *Bifidobacterium longum* (OR: 1.17, 95% CI: 1.00–1.36), we observed positive associations with CRC in IVW analysis (*p* < 0.05; Figure 2). However, only *Bilophila*, *Lachnospiraceae FCS020 group*, and *Prevotella7* obtained similar estimates in WM and MR-RAPS methods (Table 1). Leave-one-out analysis detected that the results of *Lachnospiraceae FCS020 group* and *Prevotella7* remained stable (Supplementary Figure S9).

### 3.5. Liver cancer

We noticed 11 and 13 bacterial taxa associated with HCC and ICC in IVW test, respectively (Figure 2). Six taxa, namely, *Clostridia* (OR: 3.21, 95% CI: 1.01–10.22), *Enterobacteriales* (OR: 4.52, 95% CI: 1.05–19.40), *Enterobacteriaceae* (OR: 4.52, 95% CI: 1.05–19.40), *Ruminococcus torques group* (OR: 4.79, 95% CI: 1.09–21.10), *Ruminococcus lactaris* (OR: 4.79, 95% CI: 1.46–15.74), and *Bacteroides clarus* (OR: 1.96, 95% CI: 1.09–3.53) were positively associated with HCC (*p* < 0.05). Of these, *Enterobacteriales* (OR: 5.63, 95% CI: 1.16–27.43) and *Enterobacteriaceae* (OR: 5.63, 95% CI: 1.16–27.43) were also positively correlated with ICC risk (*p* < 0.05). Additionally, *Veillonellaceae* (OR: 3.58, 95% CI: 1.29–9.93), *Alistipes* (OR: 5.65, 95% CI: 1.32–24.24), *Oscillospira* (OR: 6.38, 95% CI: 1.44–28.30), and *Slackia* (OR: 3.34, 95% CI: 1.06–10.52) were also related to a higher risk of ICC (*p* < 0.05).

On the contrary, we found negative associations of *Christensenellaceae* (OR: 0.13, 95% CI: 0.03–0.57), *Christensenellaceae R 7group* (OR: 0.16, 95% CI: 0.04–0.77), *Butyricicoccus* (OR: 0.22, 95% CI: 0.06–0.79), *Parabacteroides goldsteinii* (OR: 0.43, 95% CI: 0.23–0.83), and *Lachnospiraceae bacterium 7_1_58FAA* (OR: 0.25, 95% CI: 0.10–0.63) with HCC risk (*p* < 0.05), and *Verrucomicrobia* (OR: 0.17, 95% CI: 0.05–0.59), *Anaerostipes* (OR: 0.14, 95% CI: 0.03–0.64), *Paraprevotella* (OR: 0.27, 95% CI: 0.11–0.67), *Parasutterella* (OR: 0.32, 95% CI: 0.11–0.99), *Bifidobacterium longum* (OR: 0.31, 95% CI: 0.11–0.88), *Bacteroides clarus* (OR: 0.52, 95% CI: 0.28–0.99), as well as *Bacteroides fragilis* (OR: 0.48, 95% CI: 0.28–0.83) were negatively associated with ICC risk (*p* < 0.05).

The WM and MR-RAPS analyses indicated that the associations of *Butyricicoccus* and *Ruminococcus lactaris* with HCC risk remained, and the associations of *Verrucomicrobia*, *Enterobacteriales*, *Enterobacteriaceae*, *Veillonellaceae*, *Paraprevotella*, and *Bacteroides clarus* with ICC risk remained (Table 1). Finally, stable results were achieved in *Verrucomicrobia*, *Veillonellaceae*, and *Paraprevotella* by leave-one-out analysis, whereas there were some SNPs in *Butyricicoccus*, *Ruminococcus lactaris, Enterobacteriales*, *Enterobacteriaceae*, and *Bacteroides clarus* with dominant effects on the causal estimations (Supplementary Figures S10, S11).

### 3.6. Pancreatic cancer

Genetically predicted *Bacillales* (OR: 1.67, 95% CI: 1.23–2.26), *Defluviitaleaceae UCG011* (OR: 1.63, 95% CI: 1.01–2.64), *Sutterella* (OR: 2.45, 95% CI: 1.38–4.37), and *Bacteroides plebeius* (OR: 1.41, 95% CI: 1.07–1.85) were associated with higher pancreatic cancer risk in IVW method (*p* < 0.05; Figure 2). Differently, *Ruminococcaceae* (OR: 0.49, 95% CI: 0.26–0.93), *Eggerthella* (OR: 0.63, 95% CI: 0.43–0.93), *Lachnospiraceae UCG004* (OR: 0.52, 95% CI: 0.29–0.92), *Alistipes putredinis* (OR: 0.38, 95% CI: 0.18–0.82), *Flavonifractor plautii* (OR: 0.57, 95% CI: 0.40–0.82), *Eubacterium hallii* (OR: 0.61, 95% CI: 0.46–0.83), *Dialister invisus* (OR: 0.51, 95% CI: 0.33–0.79), *Parasutterella excrementihominis* (OR: 0.70, 95% CI: 0.51–0.96), and *Bacteroides stercoris* (OR: 0.62, 95% CI: 0.40–0.97) were related to lower pancreatic cancer risk (*p* < 0.05; Figure 2). As shown in Table 1, the WM and MR-RAPS methods revealed similar results in the potential causal association analysis of *Bacillales*, *Eggerthella*, *Sutterella*, *Flavonifractor plautii*, and *Eubacterium hallii* with pancreatic cancer. Furthermore, leave-one-out test indicated that except for *Eggerthella*, no SNPs with dominant effects were identified in other genetic predictions (Supplementary Figure S12).

### 3.7. Bonferroni correction and sensitivity analysis

The Bonferroni correction indicated that phylum *Verrucomicrobia* retained a strong negative association with ICC (OR: 0.17, 95% CI: 0.05–0.59, *p* = 0.005), whereas order *Bacillales* retained a strong positive association with pancreatic cancer (OR: 1.67, 95% CI: 1.23–2.26, *p* = 0.001). The *p* values of the intercept terms of MR-Egger regression were all greater than 0.05, revealing no notable pleiotropy (Supplementary Table S2). Cochran’s Q test and MR-PRESSO detected no evidence of heterogeneity and outliers (*p* > 0.05; Supplementary Table S2).

### 3.8. Reverse MR analysis

After a series of IV screening steps, 11 esophageal cancer associated-SNPs, eight gastric cancer-associated SNPs, 24 CRC-associated SNPs, five HCC-associated SNPs, three ICC-associated SNPs, and 13 pancreatic cancer-associated SNPs were eligible IVs. For HCC and ICC, reverse MR analysis was not performed because the number of SNPs available in the outcome (gut microbiome) was less than 3 and no proxy SNPs (*R*^2^ > 0.8) were found or proxy SNPs were also unavailable in the outcome. Details of IVs for reverse MR are listed in Supplementary Table S3.

We found that esophageal cancer, gastric cancer, CRC, and pancreatic cancer were associated with 9, 12, 23, and 20 microbial taxa, respectively, by the IVW method (Supplementary Table S4). Among them, the associations of esophageal cancer with two microbial taxa, gastric cancer with four microbial taxa, CRC with six microbial taxa, and pancreatic cancer with five microbial taxa remained stable in WM and MR-RAPS methods (Supplementary Table S5). In particular, esophageal cancer was negatively correlated with *Ruminococcaceae UCG004* and *Dorea longicatena*, CRC was negatively correlated with *Lentisphaerae*, *Lentisphaeria*, *Victivallales*, *Clostridiaceae1*, *Victivallaceae* and *Bacteroides ovatus*, as well as pancreatic cancer was negatively correlated with *Lachnospiraceae* and *Eubacterium eligens group*. Conversely, gastric cancer was positively associated with *Candidatus Soleaferrea*, *Barnesiella intestinihominis*, *Parabacteroides unclassified*, and *Clostridium leptum*, as well as pancreatic cancer was positively associated with *Alistipes finegoldii*, *Pseudoflavonifractor capillosus*, and *Bacteroides fragilis*. The Bonferroni correction revealed that CRC retained a strong negative association with family *Clostridiaceae1* (OR: 0.91, 95% CI: 0.86–0.96, *p* = 0.001).

## 4. Discussion

Through MR analysis of 297 microbial taxa and six GI cancers (esophageal cancer, gastric cancer, CRC, HCC, ICC, and pancreatic cancer), we identified potential causal associations between 21 bacterial taxa and GI cancers (values of *p* < 0.05 in all three MR methods), and a strong potential causality was identified in two of them by Bonferroni correction (phylum *Verrucomicrobia* and order *Bacillales*). Reverse MR indicated that GI cancer was associated with 17 microbial taxa in all three MR methods, among them, a strong inverse association between CRC and family *Clostridiaceae1* was identified by Bonferroni correction. To our knowledge, this work comprehensively reveals the potential causality between gut microbiota and GI cancer using MR analysis for the first time.

We discovered that *Howardella* and *Roseburia unclassified* were positively related to gastric cancer, and *Lachnospiraceae FCS020 group* was positively associated with CRC in all three MR methods. *Howardella, Roseburia unclassified*, and *Lachnospiraceae FCS020 group* belong to the family *Lachnospiraceae*, which can produce short-chain fatty acids with an inhibitory effect on inflammation, such as acetate and butyrate, and is generally considered a beneficial bacterium (Sivaprakasam et al., 2016; Sorbara et al., 2020). However, some studies have found that the gut microbiota of patients with metabolic diseases, non-alcoholic fatty liver disease, primary sclerosing cholangitis with inflammatory bowel disease, and chronic kidney disease is enriched with *Lachnospiraceae* (Vacca et al., 2020). Results regarding the association of *Lachnospiraceae* with gastric cancer and CRC are also inconsistent (Aviles-Jimenez et al., 2014; Yang et al., 2019; Clos-Garcia et al., 2020; Yu et al., 2021). This discrepancy may be due to the different genera and species of family *Lachnospiraceae* playing different roles in diseases, and the relationship of *Lachnospiraceae* with gastric cancer and CRC requires further exploration at more nuanced levels (i.e., genus and species levels). The exact mechanism underlying the effect of *Howardella* and *Roseburia unclassified* on gastric cancer as well as *Lachnospiraceae FCS020 group* on CRC deserves validation studies. At present, there are many studies on the association between gastric microbiome and gastric cancer, while there are few studies on the association between gut microbiome and gastric cancer, especially the research using metagenomic sequencing is more limited. Therefore, the positive associations between gastric cancer and four bacterial taxa (one genus and three species) identified in our reverse MR analysis were also not found in previous observational studies.

The three MR analysis methods also identified that *Bilophila* and *Prevotella7* were negatively and positively correlated with CRC risk, respectively. *Bilophila wadsworthia*, one species of genus *Bilophila*, can produce hydrogen sulfide, which has a promoting effect on CRC (Yazici et al., 2017). However, the association between *Bilophila* and CRC has obtained conflicting results in observational studies (Feng et al., 2015; Yang et al., 2021). The function and mechanism of *Bilophila* on CRC warrant verification studies. Previous studies have indicated that the increase of *Prevotella* abundance is related to the deficiency of the anti-inflammatory cytokine IL-10 and that inflammation is a recognized driver of colorectal carcinogenesis (Janney et al., 2020). Ternes et al. (2022) also found that *Prevotella7* was enriched in fecal samples from CRC patients. The comparison of the results of our study with those of previous studies on gut microbiota and CRC is shown in Supplementary Table S6. Furthermore, reverse MR demonstrated that CRC was negatively related to *Lentisphaerae*, *Lentisphaeria*, *Victivallales*, *Clostridiaceae1*, *Victivallaceae*, and *Bacteroides ovatus*. Only two microbial taxa, *Clostridiaceae1* and *Bacteroides ovatus*, each had an observational study consistent with our findings (Supplementary Table S6).

*Butyricicoccus*, a butyrate producer, was detected to be inversely associated with HCC in our study, which remained stable across the three MR methods. Consistently, several observational studies also found that *Butyricicoccus* abundance was reduced in HCC patients (Ren et al., 2019; Lapidot et al., 2020). Supplementary Table S7 showed the comparison of our results with previous studies on gut microbiota and HCC. We also observed negative associations of *Verrucomicrobia* and *Paraprevotella* with ICC, and positive correlations of *Enterobacteriales*, *Enterobacteriaceae*, and *Veillonellaceae* with ICC. There are currently few studies concentrating on the relationship between intestinal microbiota and ICC, but some prior studies indicated that *Verrucomicrobia* and *Paraprevotella* were decreased in HCC patients, while *Enterobacteriales*, *Enterobacteriaceae*, and *Veillonellaceae* were enriched (Ponziani et al., 2019; Huang et al., 2020; Lapidot et al., 2020). Similarly, our study also found that *Enterobacteriales* and *Enterobacteriaceae* were associated with increased HCC risk in IVW method. In addition, we found a positive association between *Ruminococcus lactaris* and HCC and a negative association between *Bacteroides clarus* and ICC, which have not been reported in previous studies. We could not yet prove the mechanisms of the effects of these bacterial taxa on liver cancer, as our study mainly focused on correlation analysis. Future mechanism explanation studies are needed.

Our study also suggested that *Bacillales* and *Sutterella* were potentially causally related to increased pancreatic cancer risk, while *Eggerthella, Flavonifractor plautii*, and *Eubacterium hallii* were associated with decreased pancreatic cancer risk. The comparison of our results with previous studies on gut microbiota and pancreatic cancer is presented in Supplementary Table S8. *Sutterella* is a pro-inflammatory bacterium (Hiippala et al., 2016), and accumulating studies have suggested that gut microbiota may influence pancreatic carcinogenesis by modulating inflammatory and immune responses (Meng et al., 2018). *Eggerthella* is also considered a pro-inflammatory genus (Nikolova et al., 2021), but one study found that obese individuals had a lower relative proportion of *Eggerthella* compared to non-obese individuals (Pinart et al., 2022), and obesity significantly increased the risk of pancreatic cancer. Similarly, another study found that *Flavonifractor plautii* was more abundant in non-obese compared with obese individuals (Kasai et al., 2015). This seems to suggest the beneficial roles of *Eggerthella and Flavonifractor plautii*. The mechanism underlying the influence of *Eggerthella* and *Flavonifractor plautii* on pancreatic cancer deserves to be studied. *Eubacterium hallii* is one of the major producers of butyrate in the human gut with health-promoting effects (Hiippala et al., 2018). Observational studies also found that the abundance of *Eubacterium hallii* was higher in healthy controls than in pancreatic cancer patients (Matsukawa et al., 2021; Zhou et al., 2021). As for order *Bacillales*, further studies are warranted to clarify the functional significance of specific species and strains for pancreatic cancer. Besides, among the five microbial taxa found to be affected by pancreatic cancer in reverse MR, only the inverse associations of pancreatic cancer with *Lachnospiraceae* and *Eubacterium eligens group* were also found in previous observational studies (Supplementary Table S8).

In addition, some gut bacteria including *Oxalobacteraceae*, *Oxalobacter*, and *Ruminococcaceae UCG010* were found to be positively correlated with esophageal cancer in all three MR analysis methods. Reverse MR indicated that esophageal cancer was negatively correlated with *Ruminococcaceae UCG004* and *Dorea longicatena*. At present, little is known about the relevance between intestinal microbiota and esophageal cancer, and the association of *Oxalobacteraceae*, *Oxalobacter, Ruminococcaceae UCG010, Ruminococcaceae UCG004, and Dorea longicatena* with esophageal cancer has not been reported. Therefore, our study provided a new direction to unravel the role of gut microflora in esophageal cancer, and the mechanism of these bacteria in esophageal cancer requires further exploration.

The major advantage of our study is that we comprehensively analyzed the potential causalities of 297 microbial taxa and six GI cancers using two-sample MR method. Using MR method to investigate the association between gut microbiota and GI cancer has the following advantages. First, according to Mendel’s Laws of Inheritance, alleles are randomly allocated among the descendants, similar to randomization in randomized controlled trials (Zheng et al., 2017). In addition, genotypes are fixed at conception and cannot be modified by diseases (Zheng et al., 2017). Thus, causal inference is unlikely to be influenced by reverse causality and confounders. Second, the two-sample MR is based on publicly available large-scale GWAS summary-level data without additional experimental costs. However, our study has several limitations. First, since SNPs with *p* < 5 × 10^−8^ were too limited, we selected SNPs with *p* < 1 × 10^−5^ as IVs. To obtain reliable IVs, we performed a series of IV screening steps, including excluding SNPs with *F* < 10 to avoid weak IVs bias and searching all SNPs in PhenoScanner to avoid confounding effects. Second, although 297 microbial taxa were included in our analysis, the potential causal associations of many other microbial taxa with GI cancers were not explored. Especially at the species level, only 101 species were included in our study. Third, this MR is a correlation analysis of gut microbiota and GI cancer without explaining the mechanism. Fourth, the MR analysis may be affected by potential pleiotropy. Of note, all exposures in our MR analysis had 3 or more IVs, which may mitigate the impact of potential pleiotropy to some extent, because pleiotropy is unlikely to generate the same association for different IVs (Davey Smith and Hemani, 2014). Fifth, the variances of some microbial taxa explained by the genetic IVs were small, so estimates of the associations might be hampered by limited statistical power. Sixth, the participants in present study were mostly of European ancestries and only a small number of intestinal microbiome data were drawn from other ethnicities, which were less affected by ethnic bias. However, this may limit the applicability of the results to other populations.

In conclusion, this MR study demonstrates that gut microbiota has potential causal impacts on GI cancer. Our results probably offer useful biomarkers for non-invasive early diagnosis of GI cancer. In addition, our results imply that modulation of intestinal microbiome may be a potential intervention target for GI cancer prevention.

## Data availability statement

The original contributions presented in the study are included in the article/Supplementary material, further inquiries can be directed to the corresponding authors.

## Author contributions

QS, GC, and YW designed the research. QS, CJ, ZB, YY, JiW, JuW, JZ, YC, and HZ collected and analyzed the data. QS drafted the manuscript. GC and YW supervised the study and revised the manuscript. All authors contributed to the article and approved the submitted version.

## Funding

This study was jointly supported by the National Natural Science Foundation of China (No. 81703310, 81772628, and 82072685) and the Science and Technology Plan Project of Wenzhou (No. Y2020153).

## Conflict of interest

The authors declare that the research was conducted in the absence of any commercial or financial relationships that could be construed as a potential conflict of interest.

## Publisher’s note

All claims expressed in this article are solely those of the authors and do not necessarily represent those of their affiliated organizations, or those of the publisher, the editors and the reviewers. Any product that may be evaluated in this article, or claim that may be made by its manufacturer, is not guaranteed or endorsed by the publisher.

## Footnotes

^1^ https://shiny.cnsgenomics.com/mRnd/
