# Supplementary material for: Association between gut microbiota and gastrointestinal cancer: a two-sample bi-directional Mendelian randomization study
Source: Front Microbiol. 2023 Jul 18;14:1181328. doi: 10.3389/fmicb.2023.1181328 (PMC10390774; doi:10.3389/fmicb.2023.1181328)
Supplement: Supplementary file 2 [file Data_Sheet_2.DOCX]

**Supplemental Figures**

**Supplemental Figure S1.** Scatter plot of the association between gut microbiota and esophageal cancer. (A) *Oxalobacteraceae*; (B) *Oxalobacter*; (C) *Ruminococcaceae UCG010*.

**Supplemental Figure S2.** Scatter plot of the association between gut microbiota and gastric cancer. (A) *Howardella*; (B) *Roseburia unclassified*.

**Supplemental Figure S3.** Scatter plot of the association between gut microbiota and colorectal cancer. (A) *Bilophila*; (B) *Lachnospiraceae FCS020 group*; (C) *Prevotella7*.

**Supplemental Figure S4.** Scatter plot of the association between gut microbiota and hepatocellular carcinoma. (A) *Butyricicoccus*; (B) *Ruminococcus lactaris*.

**Supplemental Figure S5.** Scatter plot of the association between gut microbiota and intrahepatic cholangiocarcinoma. (A) *Verrucomicrobia*; (B) *Enterobacteriales*; (C) *Enterobacteriaceae*; (D) *Veillonellaceae*; (E) *Paraprevotella;* (F) *Bacteroides clarus*.

**Supplemental Figure S6.** Scatter plot of the association between gut microbiota and pancreatic cancer. (A) *Bacillales*; (B) *Eggerthella*; (C) *Sutterella;* (D) *Flavonifractor plautii;* (E) *Eubacterium hallii*.

**Supplemental Figure S7.** Leave-one-out sensitivity analysis for the association between genetically predicted gut microbiota and esophageal cancer. (A) *Oxalobacteraceae*; (B) *Oxalobacter*; (C) *Ruminococcaceae UCG010*.

**Supplemental Figure S8.** Leave-one-out sensitivity analysis for the association between genetically predicted gut microbiota and gastric cancer. (A) *Howardella;* (B) *Roseburia unclassified.*

**Supplemental Figure S9.** Leave-one-out sensitivity analysis for the association between genetically predicted gut microbiota and colorectal cancer. (A) *Bilophila*; (B) *Lachnospiraceae FCS020 group*; (C) *Prevotella7*.

**Supplemental Figure S10.** Leave-one-out sensitivity analysis for the association between genetically predicted gut microbiota and hepatocellular carcinoma. (A) *Butyricicoccus*; (B) *Ruminococcus lactaris*.

**Supplemental Figure S11.** Leave-one-out sensitivity analysis for the association between genetically predicted gut microbiota and intrahepatic cholangiocarcinoma. (A) *Verrucomicrobia*; (B) *Enterobacteriales*; (C) *Enterobacteriaceae*; (D) *Veillonellaceae*; (E) *Paraprevotella;* (F) *Bacteroides clarus*.

**Supplemental Figure S12.** Leave-one-out sensitivity analysis for the association between genetically predicted gut microbiota and pancreatic cancer. (A) *Bacillales*; (B) *Eggerthella*; (C) *Sutterella;* (D) *Flavonifractor plautii;* (E) *Eubacterium hallii*.


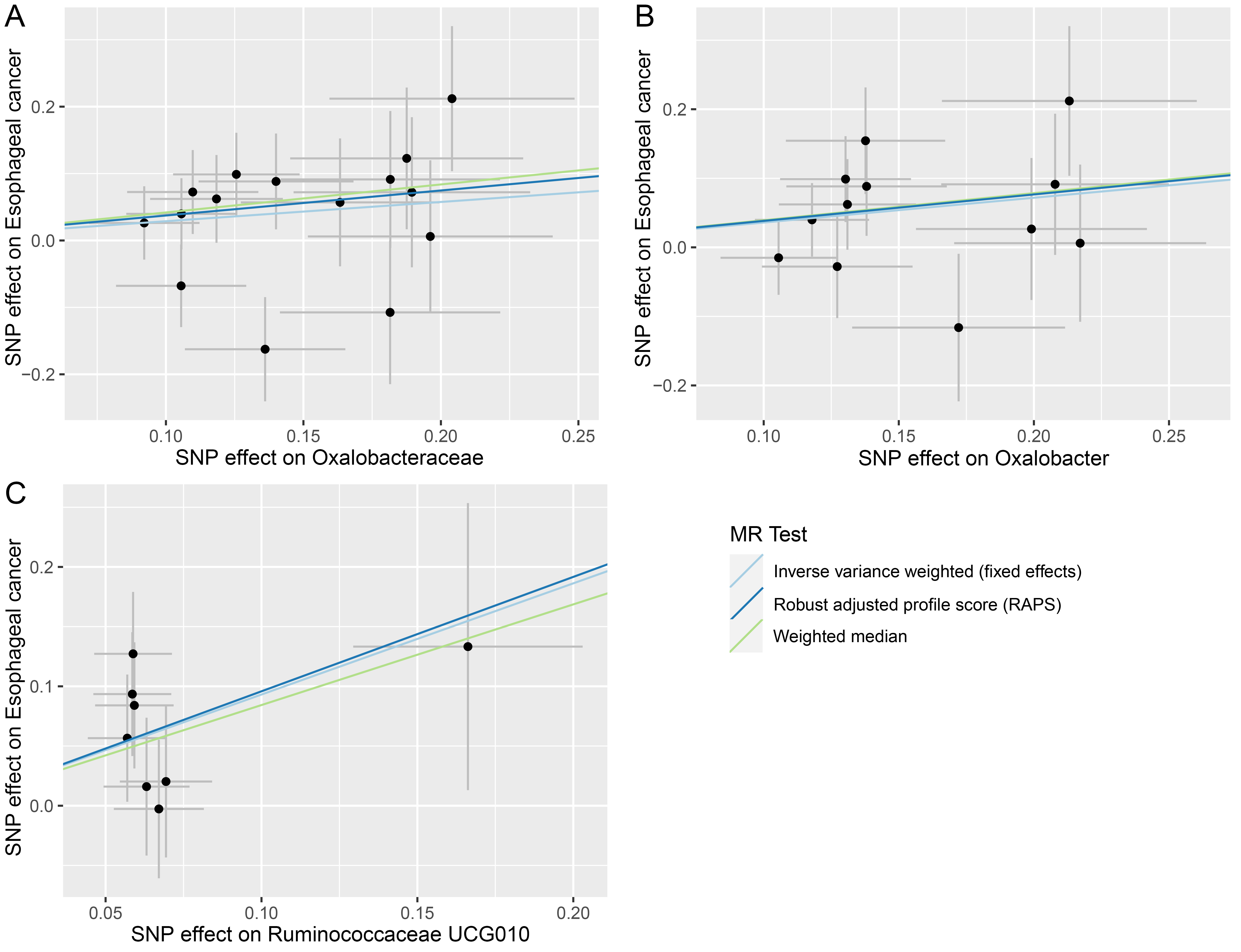


**Supplemental Figure S1.** Scatter plot of the association between gut microbiota and esophageal cancer. (A) *Oxalobacteraceae*; (B) *Oxalobacter*; (C) *Ruminococcaceae UCG010*.


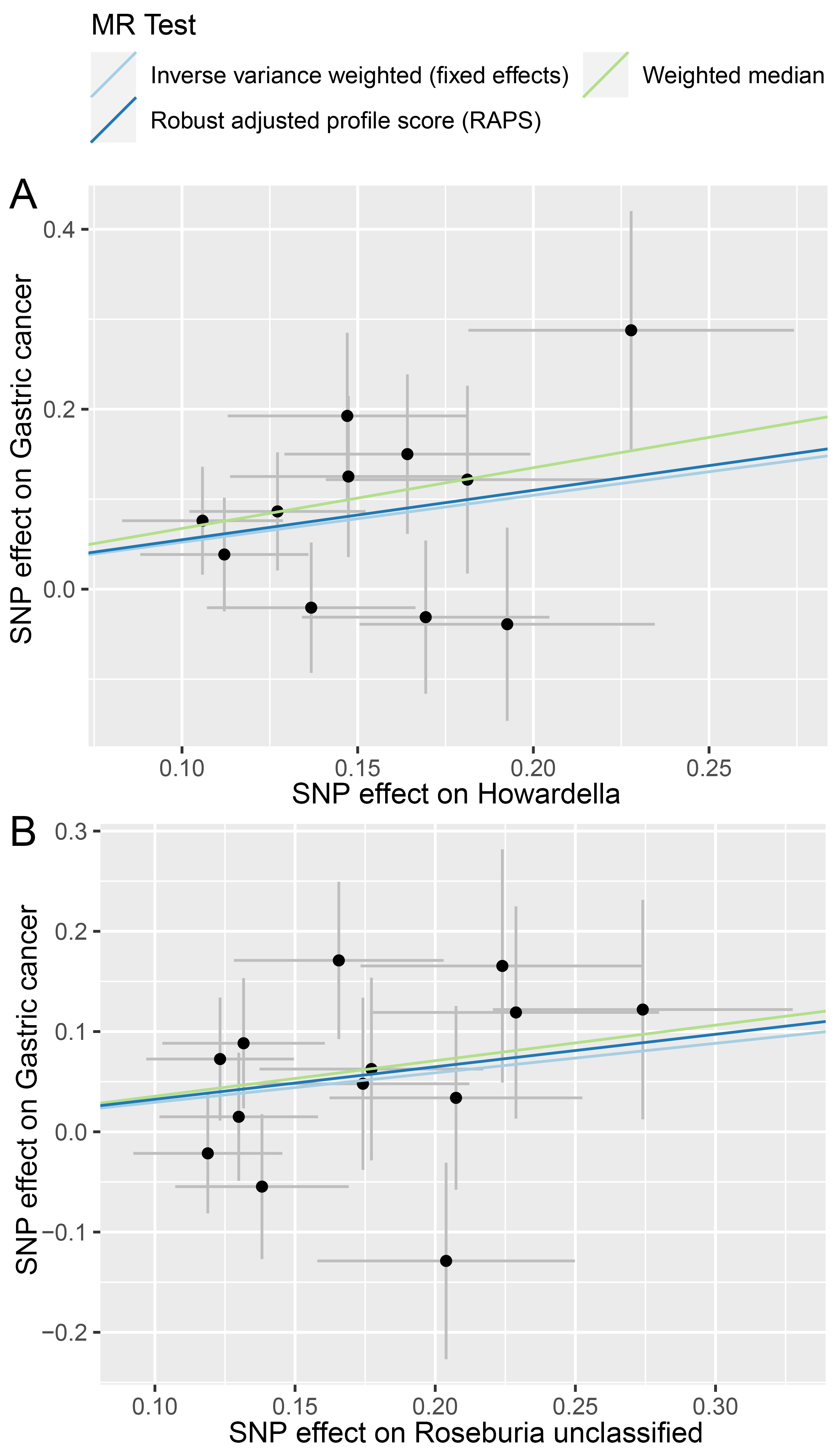


**Supplemental Figure S2.** Scatter plot of the association between gut microbiota and gastric cancer. (A) *Howardella;* (B) *Roseburia unclassified*.


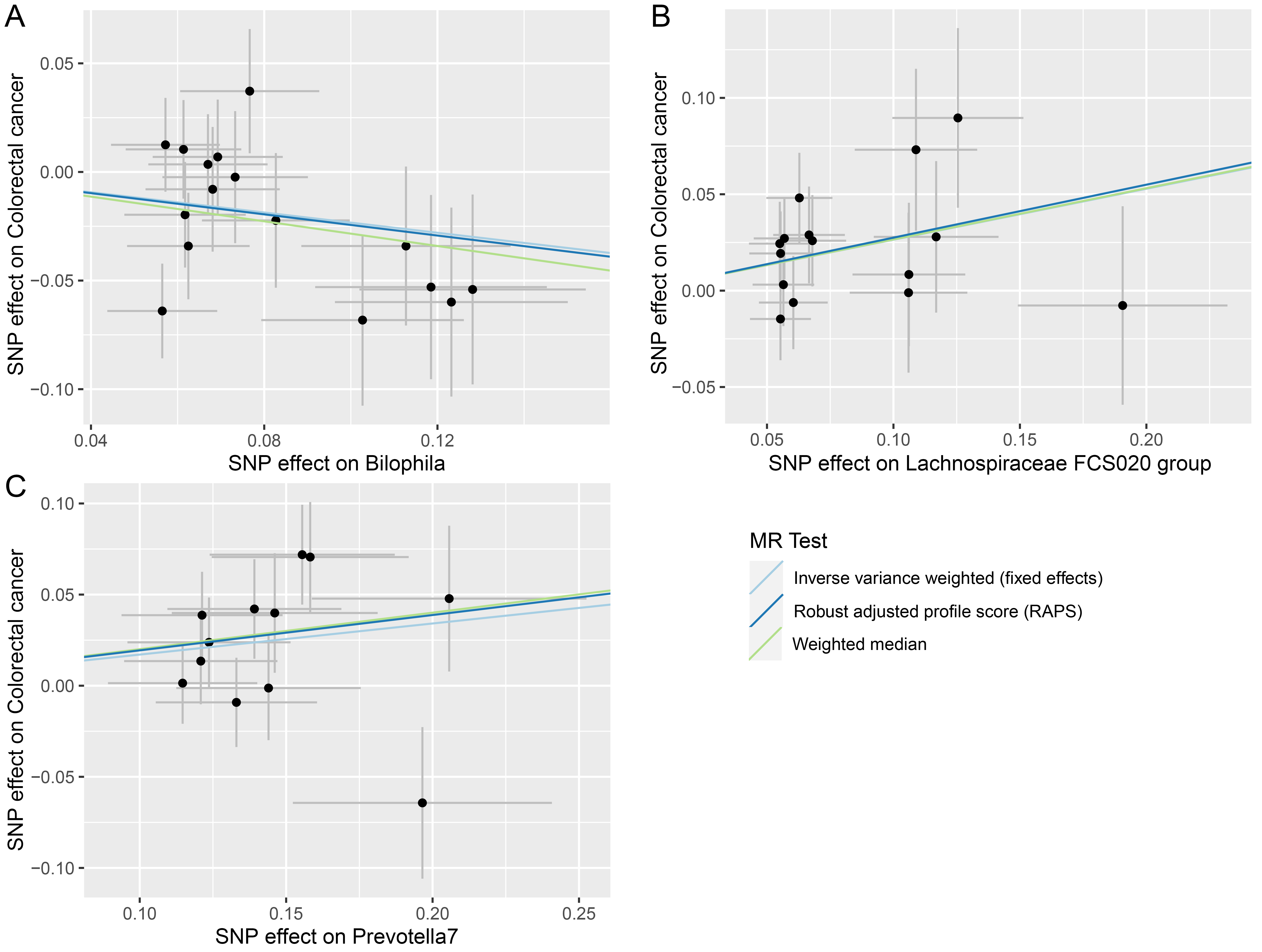


**Supplemental Figure S3.** Scatter plot of the association between gut microbiota and colorectal cancer. (A) *Bilophila*; (B) *Lachnospiraceae FCS020 group*; (C) *Prevotella7*.


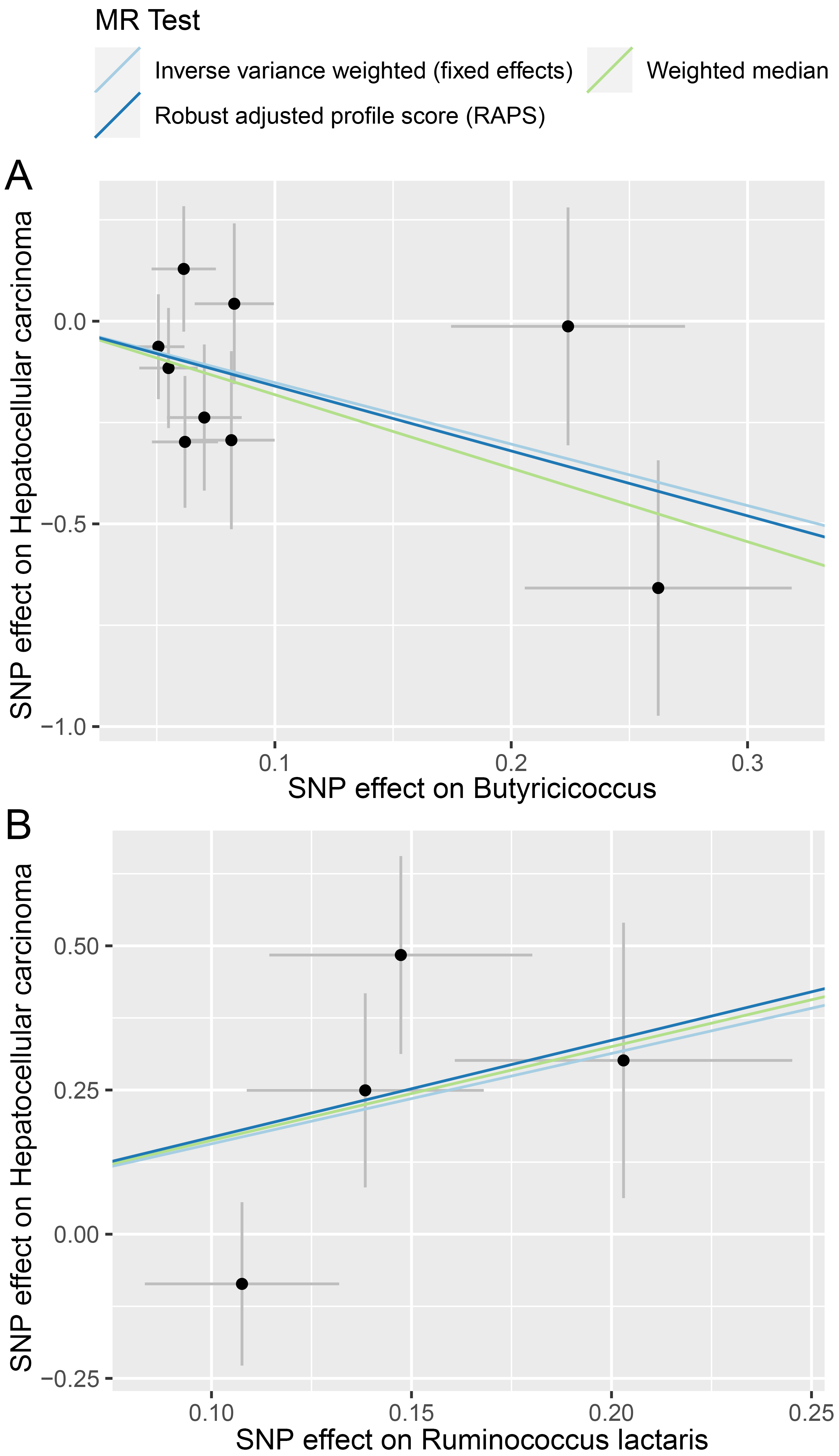


**Supplemental Figure S4.** Scatter plot of the association between gut microbiota and hepatocellular carcinoma. (A) *Butyricicoccus;* (B) *Ruminococcus lactaris.*


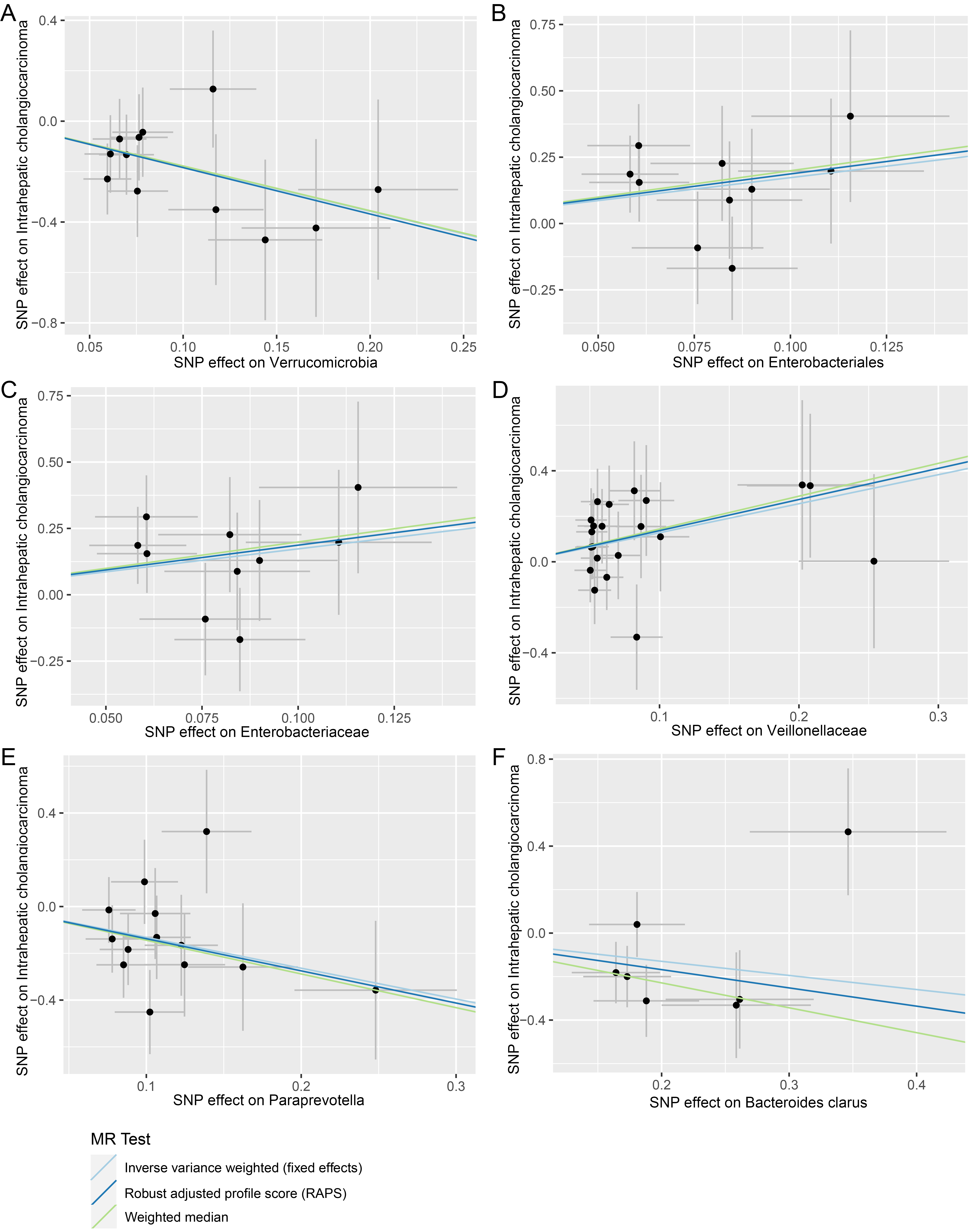


**Supplemental Figure S5.** Scatter plot of the association between gut microbiota and intrahepatic cholangiocarcinoma. (A) *Verrucomicrobia*; (B) *Enterobacteriales*; (C) *Enterobacteriaceae*; (D) *Veillonellaceae*; (E) *Paraprevotella;* (F) *Bacteroides clarus*.


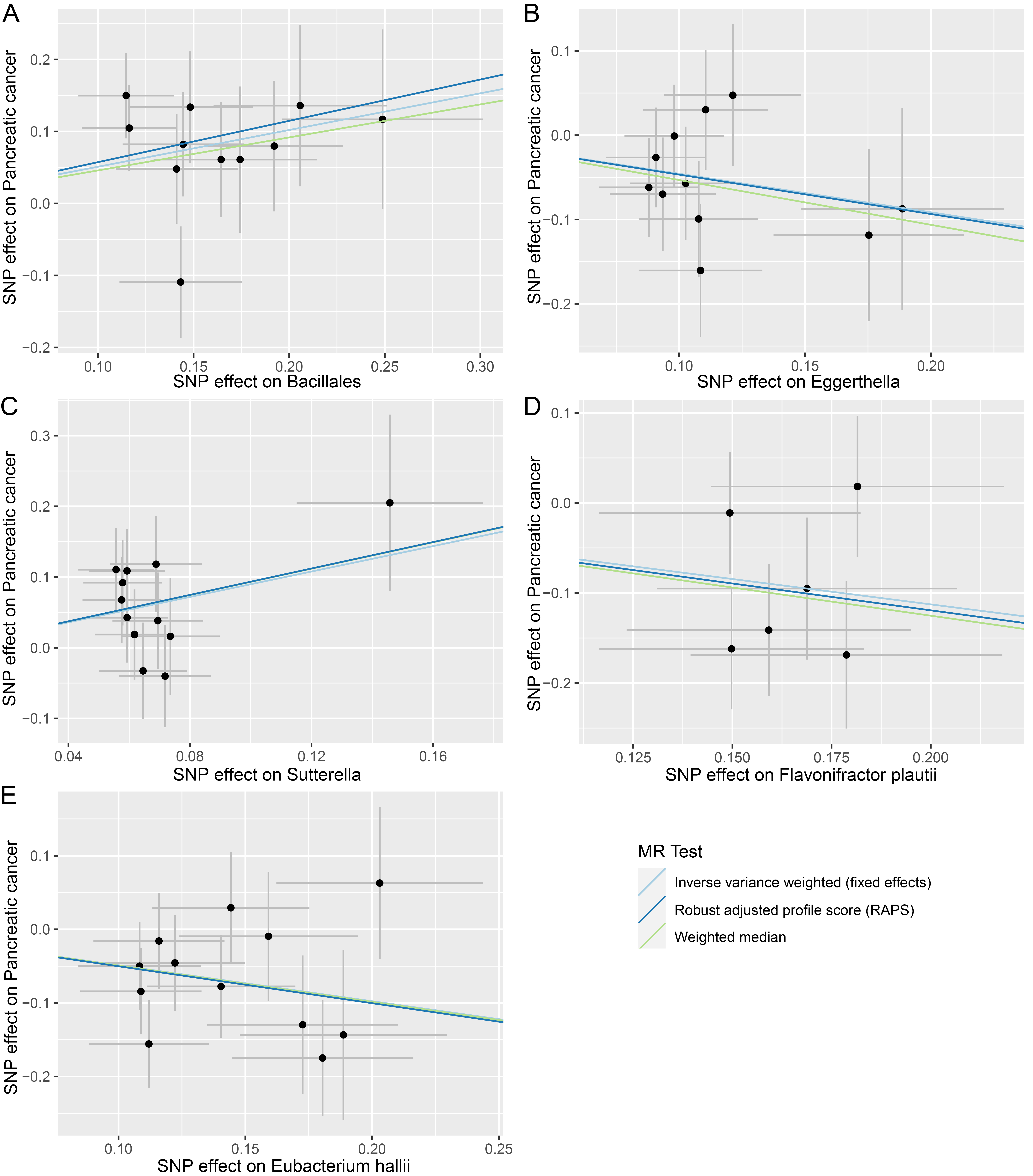


**Supplemental Figure S6.** Scatter plot of the association between gut microbiota and pancreatic cancer. (A) *Bacillales*; (B) *Eggerthella*; (C) *Sutterella;* (D) *Flavonifractor plautii;* (E) *Eubacterium hallii*.


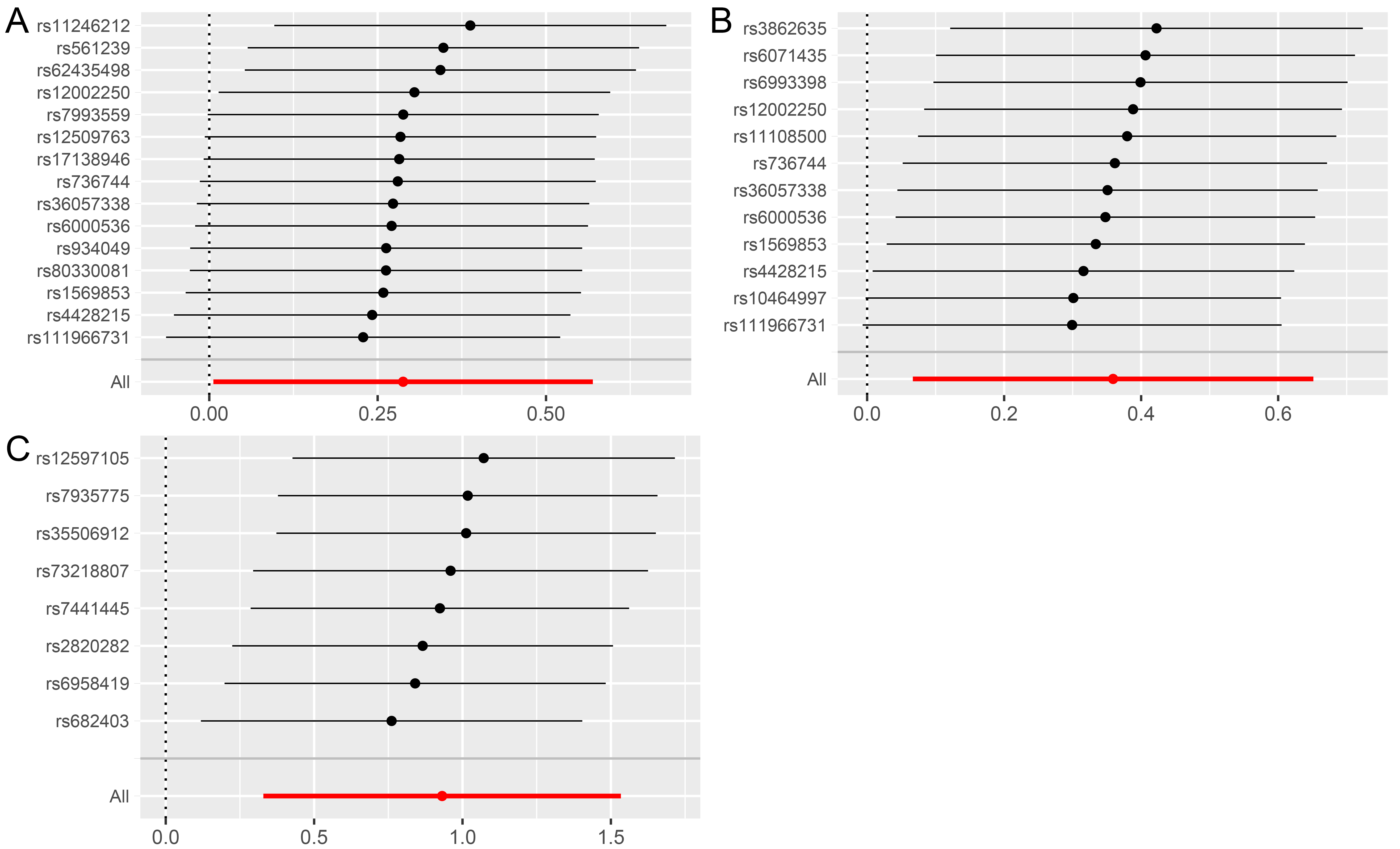


**Supplemental Figure S7.** Leave-one-out sensitivity analysis for the association between genetically predicted gut microbiota and esophageal cancer. (A) *Oxalobacteraceae*; (B) *Oxalobacter*; (C) *Ruminococcaceae UCG010*.


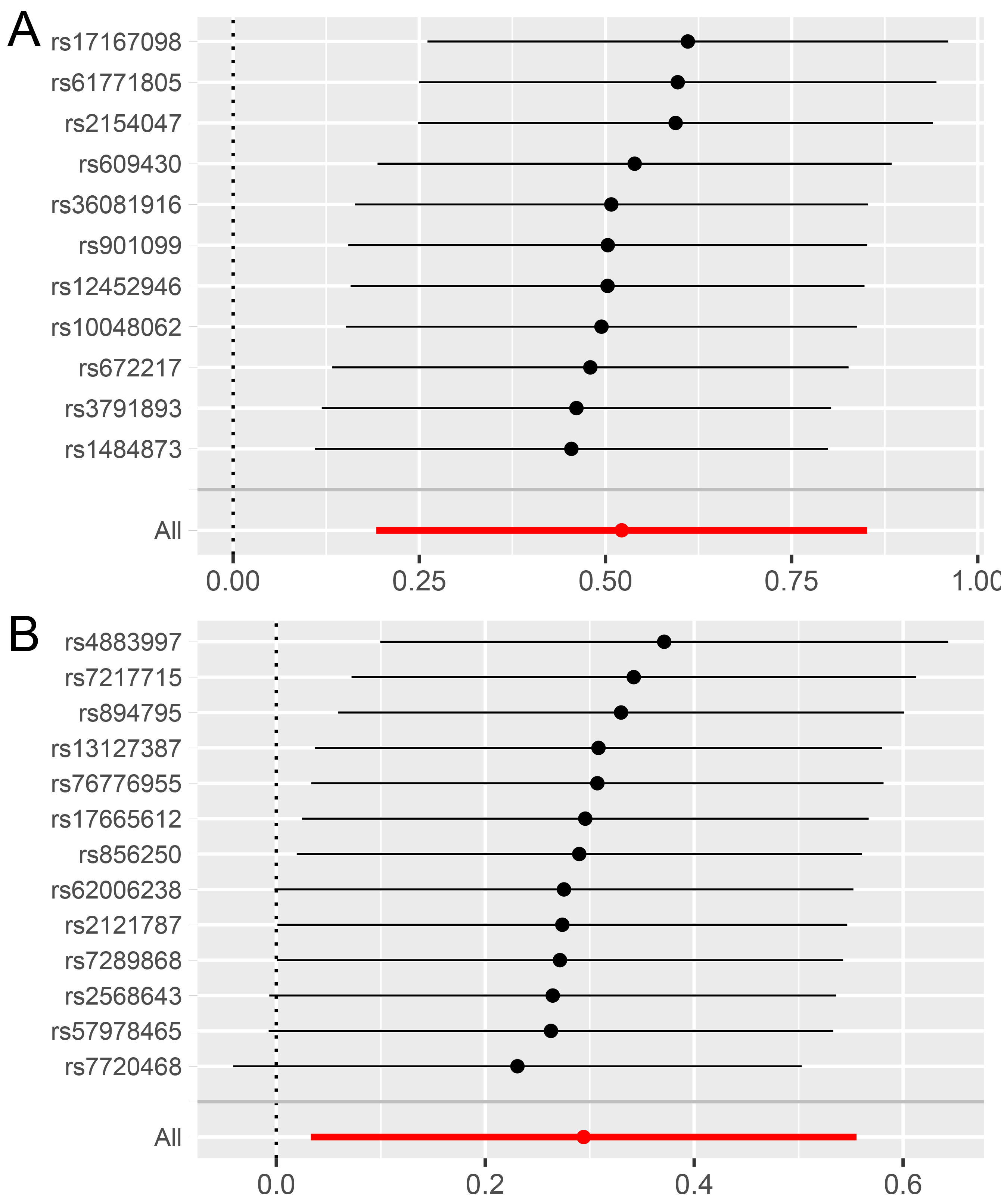


**Supplemental Figure S8.** Leave-one-out sensitivity analysis for the association between genetically predicted gut microbiota and gastric cancer. (A) *Howardella;* (B) *Roseburia unclassified.*


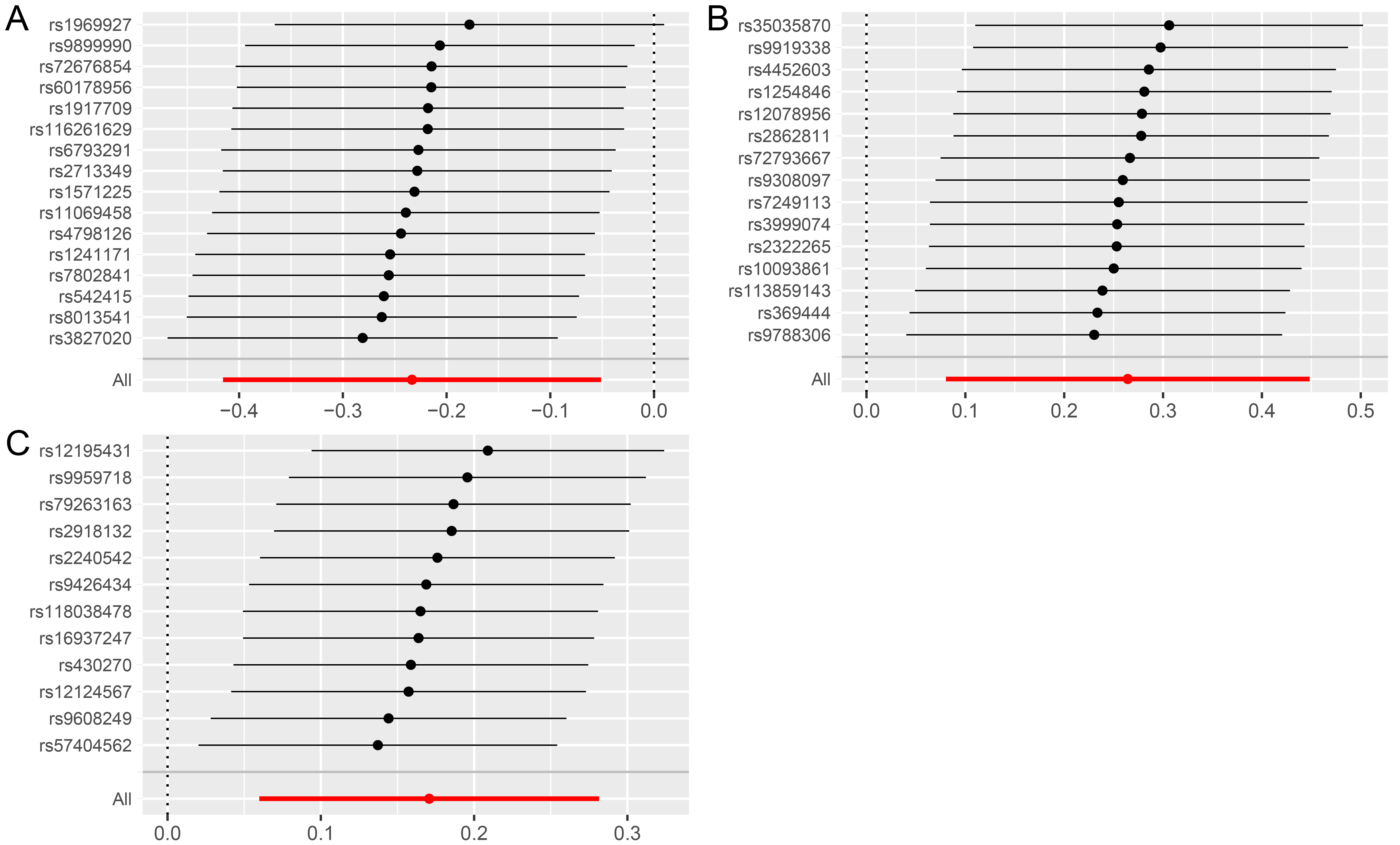


**Supplemental Figure S9.** Leave-one-out sensitivity analysis for the association between genetically predicted gut microbiota and colorectal cancer. (A) *Bilophila*; (B) *Lachnospiraceae FCS020 group*; (C) *Prevotella7*.


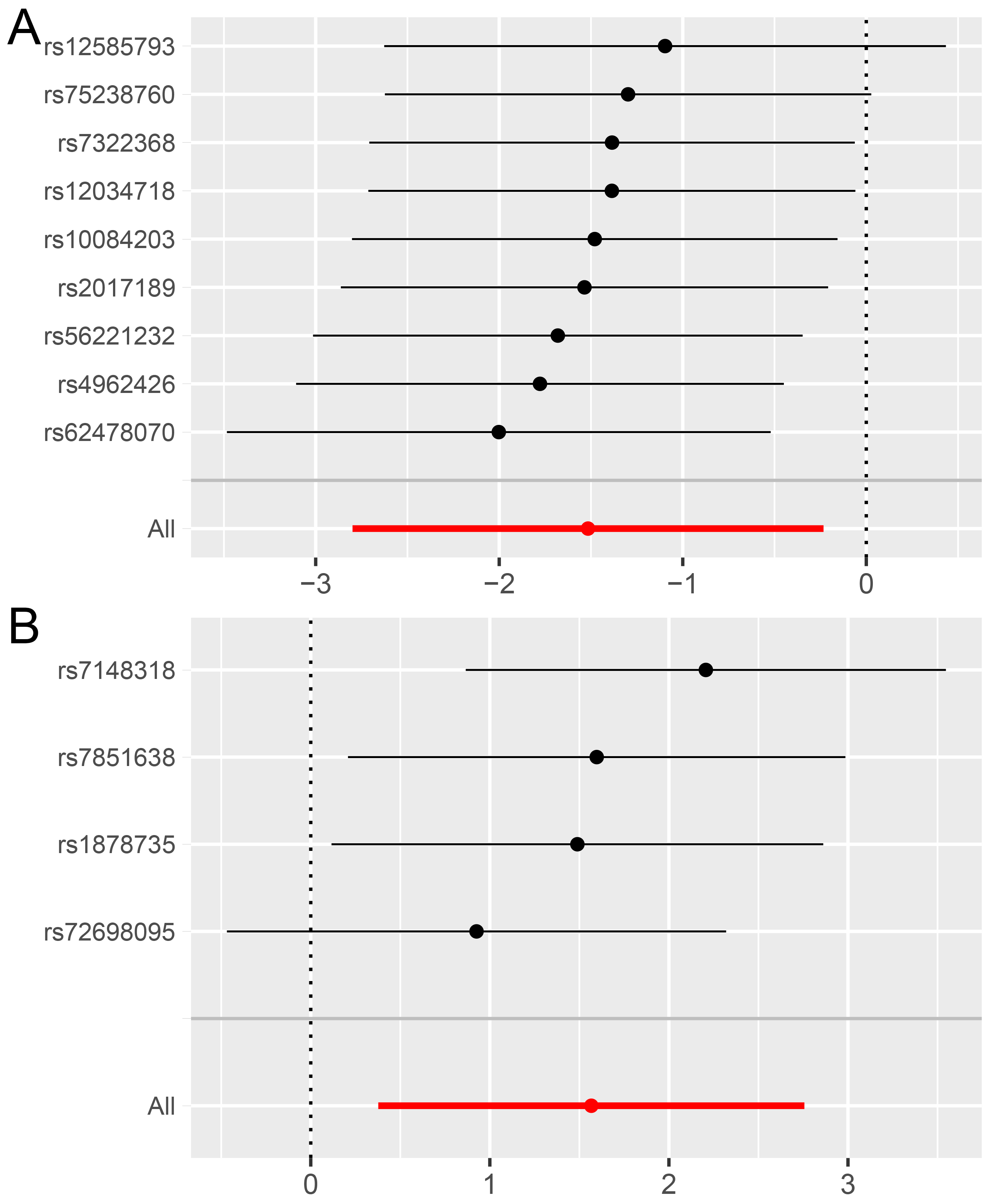


**Supplemental Figure S10.** Leave-one-out sensitivity analysis for the association between genetically predicted gut microbiota and hepatocellular carcinoma. (A) *Butyricicoccus;* (B) *Ruminococcus lactaris*.


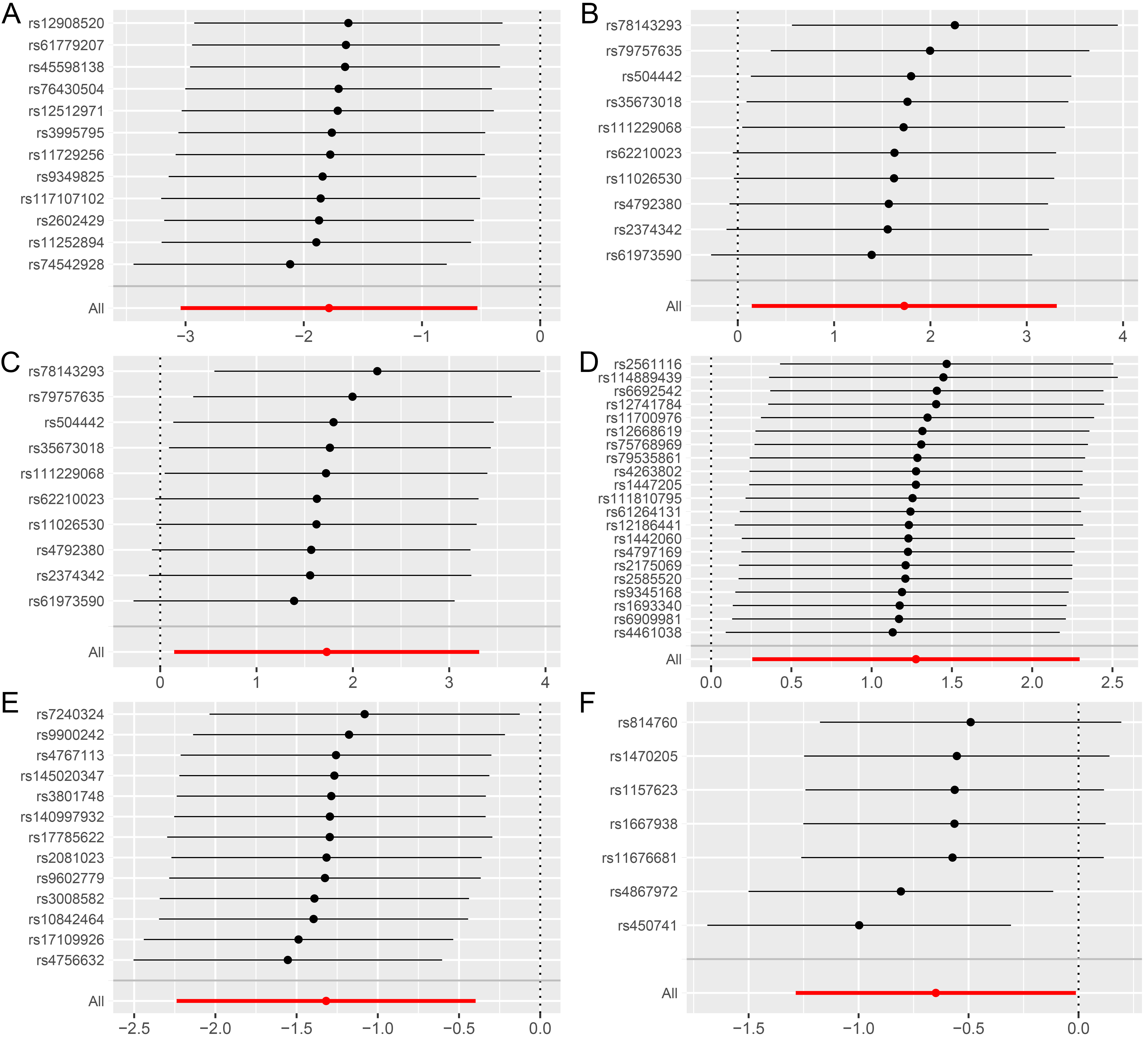


**Supplemental Figure S11.** Leave-one-out sensitivity analysis for the association between genetically predicted gut microbiota and intrahepatic cholangiocarcinoma. (A) *Verrucomicrobia*; (B) *Enterobacteriales*; (C) *Enterobacteriaceae*; (D) *Veillonellaceae*; (E) *Paraprevotella;* (F) *Bacteroides clarus*.


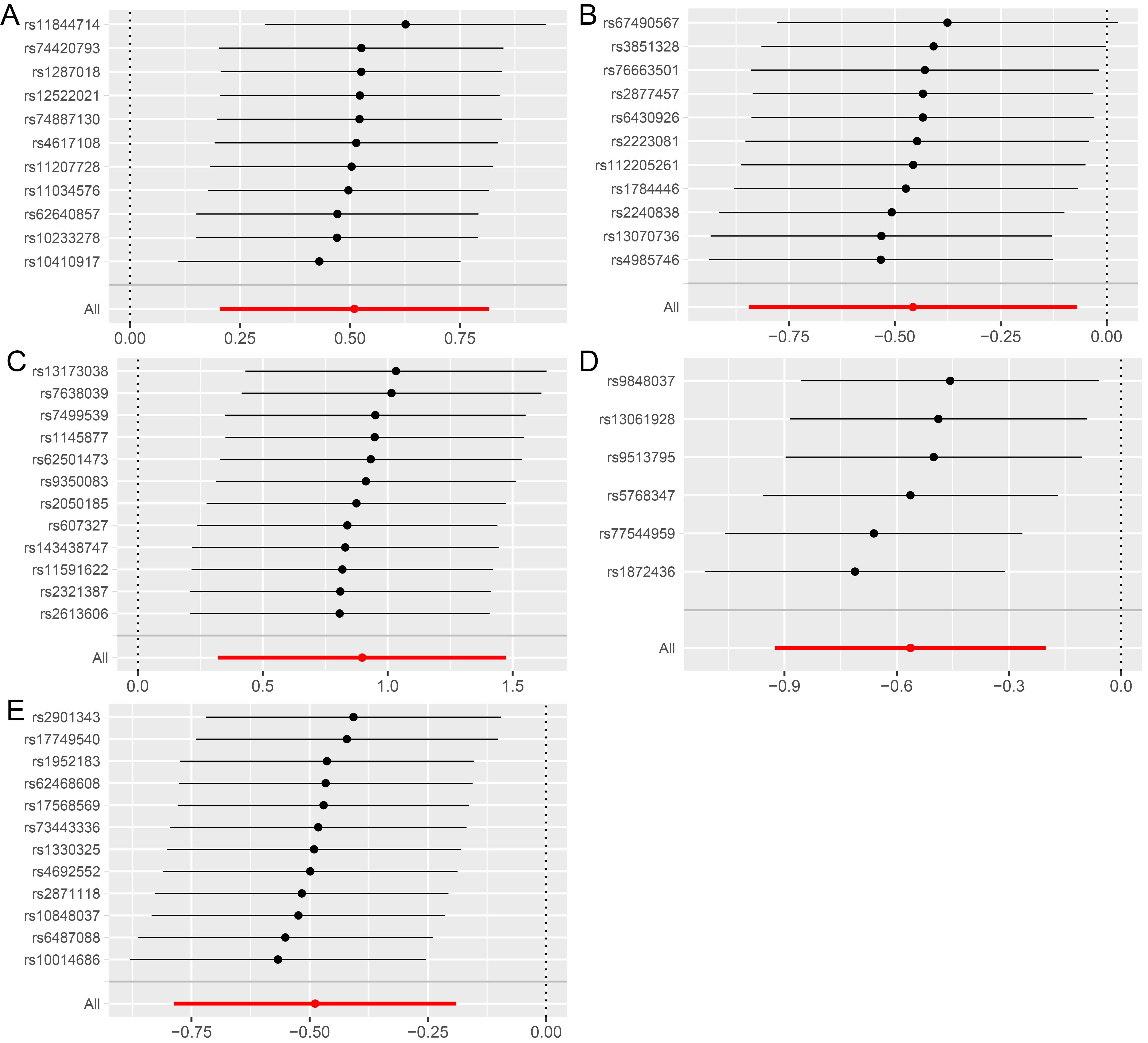


**Supplemental Figure S12.** Leave-one-out sensitivity analysis for the association between genetically predicted gut microbiota and pancreatic cancer. (A) *Bacillales*; (B) *Eggerthella*; (C) *Sutterella;* (D) *Flavonifractor plautii;* (E) *Eubacterium hallii*.
